# Chemsex and sexual risk behavior among MSM on PrEP in Stockholm, Sweden

**DOI:** 10.1016/j.dadr.2025.100367

**Published:** 2025-08-10

**Authors:** Rost Elin, Westergren Victor, Luksha Yauheni, Anna Mia Ekström, Lindberg Daniel

**Affiliations:** a Department of Infectious Diseases/Venhälsan, Södersjukhuset, Stockholm, Sweden; b Department of Global Public Health, Karolinska institutet, Stockholm, Sweden; c Department of Clinical Research and Education, Karolinska Institutet, Stockholm, Sweden; d Division of Social Work, Department of Behavior, Law and Social Science, Örebro University, Sweden

**Keywords:** Chemsex, PrEP, MSM, Sexual risk behavior, Sexualized drug use, STI, Sweden

## Abstract

**Background:**

Chemsex, typically defined as the use of specific psychoactive substances to enhance sexual experiences, has been linked to increased sexual health risks among men who have sex with men (MSM). This study examines frequency of chemsex use and associations between chemsex, risk-taking, and sexual practices among MSM on pre-exposure prophylaxis against HIV (PrEP) attending Sweden’s largest sexual health clinic.

**Methods:**

A survey among MSM on PrEP (n = 290) mapped demographics, sexualized drug use, sexual practices, and alcohol use at Venhälsan, (South General Hospital), Stockholm, Sweden.

**Results:**

18 % engaged in chemsex at least once in the past year and 49 % of respondents using chemsex reported high-risk alcohol use or alcohol dependence and 13 % classified as dependent. Most individuals on PrEP practiced unprotected sex, 42 % used condoms as passive/bottom, only 31 % as active/top. Chemsex tripled the odds of not using a condom at least 50 % of the time: adjusted for age, education, AUDIT, and being born in Sweden. Participants using chemsex were 3 times more likely, to have more than 14 temporary partners (past year) and were twice as likely to engage in group sex (82 %) compared to those not using chemsex (40 %).

**Conclusions:**

Chemsex was associated with a threefold increased risk of condomless sex and showed a significant link to alcohol dependency. While PrEP offers effective protection against HIV infection, it does not prevent sexually transmitted infections. Health professionals and social workers should identify MSM who engage in chemsex and tailor interventions to address their specific needs.

## 1 Introduction

Chemsex—the use of drugs such as methamphetamine, GHB/GBL, mephedrone, and ketamine to enhance sexual experiences—has become increasingly prevalent among men who have sex with men (MSM), raising significant public health concerns (Coyer et al., 2022, Drückler et al., 2018, Maxwell et al., 2019, Bohn et al., 2020, Scholz-Hehn et al., 2022). These substances are used to heighten sexual desire, reduce inhibitions, increase stamina and pleasure (Evers et al., 2020, Poulios et al., 2022), but are highly addictive and carry serious health risks, including dependency and injection drug use (Drückler et al., 2018, Uholyeva and Pitoňák, 2022, GBD 2016 Alcohol and Drug Use Collaborators, 2018).

Although not always included in traditional definitions of chemsex, Poppers (alkyl nitrites) and erection-enhancing drugs (EDD) such as sildenafil, have been considered separately due to their distinct pharmacological profiles and high prevalence among MSM during sexual activity. Poppers, in particular, are known to enhance sexual pleasure and reduce inhibitions by inducing smooth muscle relaxation, which can facilitate receptive anal intercourse and is associated with increased likelihood of condomless sex (Evers et al., 2020). EDDs are frequently used alongside other substances to prolong sexual encounters, and their use has also been independently associated with higher numbers of sexual partners and diagnoses (Hammoud et al., 2018).

Given these substance-specific associations with high-risk sexual behavior, and the fact that Poppers and EDDs are often used even when traditional chemsex drugs are not, they were examined both independently and as extensions of the broader chemsex definition in this study. This approach aligns with recent literature emphasizing the need to better understand substance-specific contributions to sexual health outcomes among MSM (Park et al., 2021).

Approximately one in five MSM in Europe report regular chemsex engagement (Coyer et al., 2022, Mercer et al., 2016), a trend linked to increased sexual risk-taking, unprotected sex, STI, and consent-related concerns (Scholz-Hehn et al., 2022, Maviglia et al., 2022, Amundsen et al., 2022, Maxwell et al., 2019, De La Mora et al., 2023). Beyond sexual enhancement, chemsex can serve as a coping mechanism for minority stress, HIV stigma, and internalized homophobia (Poulios et al., 2022, Dubov et al., 2018). Social settings may normalize chemsex, complicating risk recognition, especially for younger or vulnerable MSM (Maxwell et al., 2019, De La Mora et al., 2023, Evers et al., 2020), warranting a better understanding of predictors of problematic drug use in relation to sex.

In Sweden, research on chemsex remains limited. Since PrEP introduction in 2018, HIV-related anxiety has declined, but sexual risk-taking and STI rates have increased (Hansson et al., 2020). Additionally, Sweden’s zero-tolerance drug policy (SFS, 1968:649) creates legal risks for MSM engaging in chemsex.

This study explores associations between chemsex use, frequency, sexual behaviors, and risk-taking among MSM on PrEP in Sweden, with the goal of informing targeted interventions and harm reduction strategies.

## 2 Methods

The study was conducted at Sweden’s largest HIV and STI clinic for adult MSM and transgender individuals, Venhälsan at Södersjukhuset (South General Hospital), in Stockholm. Venhälsan has a strong focus on sexual and reproductive health and rights. The clinic offers PrEP for HIV prevention since October 2018 and has so far enrolled around 2500 MSM and transgender individuals at increased risk of HIV (and other STIs) of whom approximately 2000 are still on PrEP (Venhälsan Södersjukhuset, 2025).

Data were derived from patient intake forms at the clinic where the study was conducted. Questions regarding mental and sexual health, drug use, and the occurrence of violence are routinely asked at all patient visits with nurses or doctors at the clinic to identify patients in need of support from a counsellor on issues related to sexuality, mental health, and psychosocial difficulties or further referrals.

### 2.1 Recruitment of participants and data collection

The study is based on a digital, self-administered, anonymous survey, developed by an interdisciplinary team of researchers, physicians, nurses and counselors with extensive experience of infectious diseases and venerology. The technical platform was built in RedCap (Harris et al., 2009) by a research nurse (VW) and pilot tested before launch. All MSM on PrEP attending the clinic for check-ups and prescription renewals were eligible and invited to participate by the nurse or midwife who scheduled their appointment. After providing written informed consent, participants received a unique QR-code linking them to the REDCap survey. As the survey could be completed after their visit, participants, anonymity was preserved.

Assuming 80 % power and 1 % significance level, a minimum of N = 88 study participants per group was required to detect an assumed doubling of risk behaviors (number of partners/unprotected sex) among those using chemsex. Due to the lack of prior data in chemsex prevalence among Swedish MSM and allowing for a maximum of 20 % non-response rate, we targeted a total sample size of N = 300 to provide sufficient margin, should effecte sizes be smaller than anticipated.

Between September the 12th and October the 28th of 2022, 599 patients visited Venhälsan for their regular PrEP checkups. Most were invited to participate in the study; non-invitations were primarily due to staff workload and occurred at random. Among those invited, only 10 patients did not complete the questionnaire resulting in a high response rate and leaving us with 290 respondents in the final analyses.

### 2.2 Measures

#### 2.2.1 Demographics and sexual behaviour

The survey included questions on age (analysed both as a continous and categorical variable), country of birth, parents’ country of birth, education level, occupation, sexual preference, sexual orientation, and transgender experiences. Questions regarding transgender experiences included, for example, whether the participant had ever identified as transgender or undergone gender-affirming care. It also included questions on sexual practices, number of partners and instances of group sex (sex with two or more people at the same time) in the past 12 months, regular condom use (using condoms less that 50 % of sexual encounters as top and bottom respectively), and HIV risk-reducing behaviors such as the use of post-exposure prophylaxis (HIV-PEP). Additionally, we assessed satisfaction with both general life and experiences in sexual contexts.

### 2.3 Alcohol and drug use

Two international standardized instruments, previously assessed regarding validity and reliability, were used for the detection of alcohol and drug dependency: AUDIT (Alcohol Use Disorders Identification Test) and DUDIT (Drug Use Disorders Identification Test), respectively. We applied cutoffs scores for different levels of alcohol and drug dependency recommended. (Saunders et al., 1993, Berman et al., 2005). High-risk alcohol consumption was defined by an AUDIT score ≥ 8, while probable alcohol dependency was indicated by a score ≥ 15. For the multivariate analyses the AUDIT variable was summarized into three categories dividing the AUDIT score into: no or low level of high-risk alcohol use , 0–7, medium level of problematic drinking 8–14, and high likelihood of alcohol dependency: 15 and above. Likely drug dependency was defined as a DUDIT score ≥ 6. For the multivariate modelling analyses, DUDIT was summarized into three categories: 0–5 (investigation warranted), 6–24 (likely problematic drug use), 25 and above (likely dependency).

### 2.4 Chemsex

A chemsex variable was created based on respondents' reported use of specific substances. The respondents were asked if they had “used any of the following substances in connection to sex during the past 12 months”, and were provided with a list of substances including, but not limited to, common chemsex-drugs. We analyzed both a more narrow and broader definition of chemsex. The narrow definition (Chemsex) includes use of methamphetamine, mephedrone, GHB/GBL, ketamine, cocaine, ecstasy/MDMA, amphetamine, and LSD or other hallucinogens during sex. This definition incorporating not only the commonly used methamphetamine, mephedrone, GHB/GBL, and ketamine, but also cocaine, ecstasy/MDMA, amphetamine and LSD or other hallucinogens. Respondents who indicated using any of these substances during sex in the last 12 months were classified as having engaged in chemsex. Respondents were asked if they “think the use of the substance mentioned above has affected your condom use during sex in the past 12 months?” Additionally, we examined the chemsex variable even further by including Poppers and EDD use (more broad definition), as well as Poppers use alone. The broader definition (Chemsex broad) expands this to include Poppers and EDD use.

### 2.5 Data analyses

For the demographic variables, data were analyzed with descriptive statistics, using frequencies and percentages for categorical variables, median, means, and standard deviations (SD) for ordinal and continuous variables. Results are presented rounded off to full percentages in the text while one decimal is kept in the tables.

To avoid multicollinearity, we conducted a correlation analysis to assess the relationships between chemsex, AUDIT and DUDIT (Table 1). Pearson’s r was used for normally distributed, continuous variables to measure the strength and direction of the linear relationship between two variables. When data were not normally distributed or included ordinal variables, we used non-parametric measure (Spearman’s rho) instead to assess the rank-order relationship between variables. Both tests were performed to account for potential differences in data distribution and variable type, ensuring a comprehensive understanding of the associations within the data. We tested the correlation between variables and found that DUDIT was too closely correlated to the chemsex use variables to be included in the final multivariate models (Alin, 2010).

**Table 1:** Correlation between Chemsex narrow and broad variables AUDIT (n = 290) and DUDIT (n = 128).

**Correlation**	**Chemsex narrow**	**Chemsex broad**
AUDIT	.274**	.219**
DUDIT	.658**	.581**

⁎⁎ Correlation is significant at the 0.01 level (2 tailed).

Parametric analyses were conducted using linear, logistic, or ordinal regression analyses. Multiple regression analyses were used to investigate associations between chemsex and the following risk behaviors (dependent variables): irregular condom use during insertive (top) or receptive (bottom) anal sex (using condoms at least 50 % of the time), and having more than 14 anonymous partners in the past year (based on the median total number of partners (temporary+ regular). Following bivariate analyses, we applied a stepdown approach to identify important independent variables including the following: the more narrow and broad chemsex variables, AUDIT and DUDIT (analyzed in three standardized categories: low, medium and high as defined above), age as a continuous variable and as in 5 year-categories (20–24, 25–29, 30–34, 35–39, 40–44, 45 +), born in Sweden (yes/no), education divided into three categories (elementary school; upper secondary/vocational/folk high school, or college/university level), employment (employed working full-time, employed working part-time, self-employed, student, sick leave, unemployed, retired or other), sexual orientation (homosexual, bisexual, heterosexual or other), and sexual partners (regular sex partners, casual sex partners and/or anonymous sex partners in the last 12 months). Given the skewed data distribution for number of partners, where a larger proportion of the population had 15 (median value), a cutoff of 14 was used to more effectively separate individuals into distinct risk categories. This cutpoint optimizes both statistical significance and clinical relevance, enabling more precise risk assessments and interventions targeted at the high-risk group. The models were built based on correlation coefficients.

The results of the study are focused on the MSM group since only N = 7 (2.5 %) of the respondents identified as transgender, making the statistical power too low to draw any conclusions about differences at group level. We therefore chose to exclude transgender individuals from the analyses in this study.

The final multivariable models, for each of the three outcome variables, included the following covariates: Chemsex use (i.e. methamphetamine, mephedrone, GHB/GBL, and ketamine, cocaine, ecstasy/MDMA, amphetamine and LSD or other hallucinogens) (yes/no), age in 5–year categories, AUDIT (3 categories as defined above), born in Sweden (yes/no), and education (3 categories: elementary school; upper secondary/vocational/folk high school, or college/university level). The broader definition of chemsex was tested but found to be statistically non-significant in the final multivariate models and was therefore excluded from the final analysis.

The results are presented in descriptive tables, cross-tabulation tables, and correlation tables. All tests were two-tailed, and the statistical significance was set at p ≤ .05. The analyses were conducted using IBM SPSS Statistics (Version 28.0; IBM SPSS, Armonk, NY, USA).

### 2.6 Ethical considerations

The study received ethical clearance from the Swedish Ethical Review Authority (Dnr: 2022–02235–01 and Dnr 2022–04503–02). The analysis was not pre-registered and the results should be considered exploratory.

## 3 Results

The respondents to this survey, which included 290 MSM on PrEP in Stockholm, had a median age of 40 years (SD ± 10.4) and 65 % were born in Sweden. The group was socioeconomically well off, with a higher level of education and employment compared to the general population: 78 % held a university degree, and 72 % reported full-time employment. Most of the participants (91 %) defined as homosexual (Table 2).

**Table 2:** Descriptive statistics among N = 290 MSM on PrEP attending the sexual health clinic.

Characteristics		N (%) 290
Age median 40 (SD 10.4)		
Age group		
	20–24	5 (1.7)
	25–29	29 (10.0)
	30–34	59 (20.3)
	35–39	49 (16.9)
	40–44	47 (16.2)
	> 45	101 (34.8)
Born in Sweden		
	Yes	189 (65.2)
	No	101 (34.8)
Parents born in Sweden		
	Yes	135 (47.0)
	No	153 (53.0)
	Missing	2 (0.7)
Education		
	Elementary school	8 (2.8)
	Upper secondary education/vocational school/folk high school	55 (19.0)
	University/college education	225 (77.6)
	Missing	2 (0.7)
Employment		
	Employed, full-time	208 (71.7)
	Employed, part-time	12 (4.1)
	Self-employed	33 (11.4)
	Studying	20 (6.9)
	Unemployed (sick leave, retired, job seekers)	15 (5.2)
	Missing	2 (0.7)
Sexual orientation		
	Homosexual	264 (91.0)
	Bisexual	24 (8.3)
	Heterosexual	3 (1.0)
	Other	8 (2.8)
Having sex with		
	Men	258 (89.0)
	Men and women, trans and/or nonbinary	32 (11.0)
	Missing	1 (0.3)
Experience of being trans		
	Yes	7 (2.4)
	No	278 (95.8)
	Don’t want to answer	4 (1.4)
	Missing	1 (0.3)
Use of chemsex*		
	Yes	52 (17.9)
	No	238 (82.1)
Poppers use		
	With chemsex	44 (15.2)
	Only poppers	119 (41.0)
	No	127 (43.8)
Number of drugs used with chemsex*		
	0	238 (82.1)
	1	21 (7.2)
	2	15 (5.2)
	3	8 (2.8)
	4	5 (1.7)
	5	3 (1.0)
DUDIT (Drug Use Disorders Identification Test)		
	Calls for investigation** (1−5)	73 (25.2)
	There are probably drug-related issues (6−24)	33 (11.4)
	Probably addicted to drugs (>25)	39 (13.4)
AUDIT (Alcohol Use Disorders Identification Test)		
	No or low risk (0−7)	226 (77.9)
	Hazardous/harmful consumption (8−14)	58 (20.0)
	Likelihood of alcohol dependence (>15)	6 (2.1)
Sexual practice with > 1 person (group sex)		
	Yes	167 (57.6)
	No	123 (42.2)
Regular condom use during insertive anal sex (=>50 % of the times)		
	Yes	91 (31.4)
	No	164 (56.6)
Regular condom use during receptive anal sex (=>50 % of the times)		
	Yes	123 (42.4)
	No	137 (47.2)
Experience of violence in connection with sex		
	Yes	15 (5.2)
	No	275 (94.8)

⁎ Use of any of the following substances in combination with sex; Cocaine, Ecstasy, MDMA, GHB, GBL, Amphetamine, Methamphetamine, Mephedrone, Heroine or Ketamine, LSD and other hallucinogens, within the preceding 12 months

⁎⁎ Further investigation is recommended when the number of points is 1 or more "to determine whether it is illegal use" (The Swedish board of health and welfare, 2007).

**Fig. 1:**
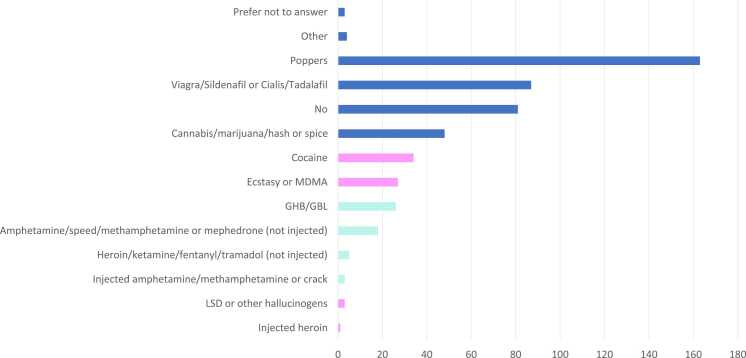
Drug use in connection with sexual activity (Chemsex) in the last 12 months.

Chemsex was relatively common and 18 % (52/290) of the repondents reported having engaged in it at least once in the past year. The use of poppers (56 %, n = 163) and erectile dysfunction drugs (30 %, n = 87) was also notably high (Fig. 1). High-risk alcohol consumption or likely dependency, based on AUDIT scores, was reported by 22 % (n = 64) of participants. Similarly, 13 % (n = 39) were likely to have drug dependency based on their DUDIT scores, and 50 % (n = 145) reported using illegal drugs in the past year. Among those who used chemsex drugs, 49 % were having high-risk alcohol use or dependency based on their AUDIT scores.

**Fig. 2:**
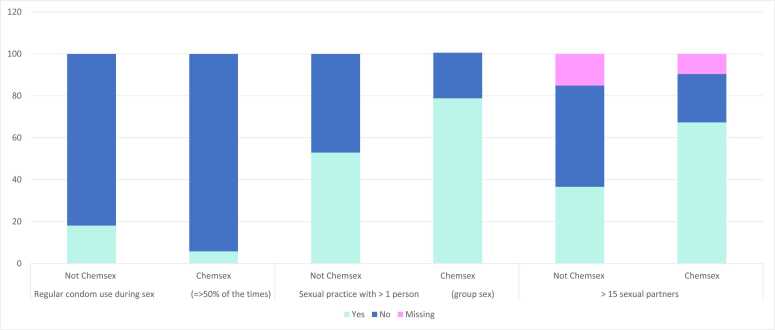
Condom use, sexual practice and partners, related to chemsex and not chemsex.

Participants used condoms more often during receptive than during insertive anal sex, 42 % (n = 123) and 31 % (n = 91) respectively. There was also a correlation between engaging in chemsex and not using condoms (Fig. 2). Among those who had engaged in chemsex in the past 12 months, only 20 % used condoms at least half of the time during insertive anal sex, and only 42 % used condoms (at least half of the time) at receptive anal sex. Chemsex more than tripled the odds of not using a condom at least 50 % of the time: OR 3.72 (CI 1.06–12.99), adjusting for age, education, AUDIT, and being born in Sweden (Table 2). Additionally, 17 % of the respondents using chemsex reported that they think use of drugs affected their condom use negatively. Among all respondents 19 % had received HIV-PEP treatment at least once.

Over the past 12 months, respondents reported a median number of 12 temporary and 3 regular sexual partners, with an overall median of 15 sex partners in total. Among the 290 MSM on PrEP who participated in the survey, 58 % (n = 167) had engaged in group sex (sex with more than one person at the same time) in the past 12 months. Group sex was twice as common among individuals who engaged in chemsex (82 %) compared to those who did not (40 %). Those who engaged in chemsex also had more temporary partners and were 3 times more likley OR 3.31 (95 % CI 1.57–6.98) of having more than 14 temporary partners in the past year, adjusted for age, education, AUDIT, and being born in Sweden (Table 2).

We examined the relationship between chemsex using both a more narrow definition and a broader definition of chemsex e.g. including poppers and EDD use and potential predictors including AUDIT, number of sexual partners, condom use practices, age, and participation in group sex in the multivariable regression model. Using the more narrow definition, both AUDIT score and number of partners was found to be significantly associated with chemsex. Alcohol use was significantly associated with chemsex (narrow) (OR = 1.23, 95 % CI 1.12–1.41). This indicates that for each one-point increase in AUDIT score, the odds of engaging in chemsex (narrow) increased by approximately 26 %. The number of sexual partners was also significantly associated with chemsex (narrow) use (OR = 1.03, 95 % CI 1.00–1.05). Condom use during sex, group sex and age was not significantly associated with chemsex use (Table 3).

**Table 3:** Regression analyses on factors associated with multiple partners, group sex and irregular condom use among N = 290.

	> 15 sexual partners (95 % CI)		Sexual practice with > 1 person (group sex)		Condom use during sex (less then 50 % of the times)	
	Crude OR (95 % CI)	Adjusted* OR (95 % CI)	Crude OR (95 % CI)	Adjusted* OR (95 % CI)	Crude OR (95 % CI)	Adjusted* OR (95 % CI)
Chemsex narrow	3.3 (1.6 – 6.8)	3.3 (1.6 – 7.0)	3.3 (1.6 – 6.8)	2.9 (1.4 – 6.2)	3.6 (1.1 – 12.1)	3.7 (1.1 – 12.9)
Age	0.9 (0.7 – 1.1)	1.0 (0.9 – 1.2)	1.0 (0.9 – 1.2)	1.1 (0.9 – 1. 2)	1.0 (0.8 – 1.2)	1.0 (0.8 – 1.2)
AUDIT	1.2 (1.1 – 1.3)	1.0 (0.9– 1.1)	1.1 (1.0 – 1.1)	1.0 (1.0– 1.1)	1.0 (0.9 – 1.1)	1.0 (0.9 – 1.1)
Born in Sweden	0.5 (0.3– 1.0)	1.1 (0.7 – 1.8)	0.7 (0.4 – 1.1)	0.8 (0.5 – 1.3)	0.7 (0.4 – 1.4)	0.8 (0.4 – 1.5)
Education	1.0 (0.5 – 1.8)	0.8 (0.5 – 1.3)	0.8 (0.5 – 1.4)	0.9 (0.6 – 1.5)	0.9 (0.4 – 1.7)	.9 (0.4 – 1.7)

⁎ The multivariable model included all variables listed in the table.

Using the broader definition of chemsex i.e. including poppers and EDD use, only AUDIT score was significantly associated with chemsex use (OR = 1.16, 95 % CI 1.01–1.32), indicating that for each one-point increase in AUDIT score, the odds of chemsex increased by approximately 16 %. While the association between chemsex use and number of partners was borderline significant (p = 0.098), the remaining variables were not significantly associated with this broader chemsex definition (Table 4). The broad definition of chemsex including poppers and EDD use was tested but found to be statistically non-significant in multivariate models and was therefore excluded from the final analysis.

**Table 4:** Logistic Regression Analysis of Factors Associated with Chemsex narrow and Chemsex broad (N = 139).

	Chemsex narrow		Chemsex broad	
	p-valu	OR (95 % CI)	p-value	OR (95 % CI)
AUDIT score	< .001	1.257 (1.122–1.409)	.033	(1.012–1.319)
Number of partners	.024	1.027 ([1.004–1.051)	.098	(0.991–1.108)
Condom use during sex (<50 % of the times)	.998	70508–2999,280 (0.720–6.499)	.169	(0.720–6.499)
Group sex	0.499	2.292 (0.207–25.420)	.724	(0.065–6.660)
Age (continues)	.054*	1.048 (0.999–1.100)	.158	(0.989–1.074)

Note: OR =  Odds Ratio; CI =  Confidence Interval; AUDIT =  Alcohol Use Disorders Identification Test

⁎ borderline significant

The sole use of poppers did not seem to have an impact on the number of temporary partners after adjustment for age, AUDIT, being Swedish born or not and educational level. There was no correlation between using poppers and riskier sexual behavior (Table 4).

## 4 Discussion

In this survey, which we believe is representative for MSM on PrEP in Stockholm, Sweden, 18 % had engaged in chemsex in the past 12 months. Chemsex was significantly associated with higher consumption of alcohol, less frequent use of condom during anal sex, increased instances of group sex, and more casual sexual partners. However, there was no significant correlation between the key outcomes (number of casual partners, condomless sex, exposure to sexual violence), and the use of poppers, or alcohol addiction (high AUDIT score alone) after adjusting for confounding from chemsex use and sociodemographic factors.

The proportion of respondents in this survey who engage in chemsex is consistent with previous international on MSM and chemsex research from the Netherlands, and in Britain (Drückler et al., 2018, Coyer et al., 2022, Mercer et al., 2016). The appeal of chemsex lies in its ability to heighten sexual desire, lower inhibitions, increase endurance, and enhance physical and emotional pleasure (Evers et al., 2020, Evers et al., 2020, Poulios et al., 2022). However, especially methamphetamine, amphetamine, mephedrone, and GHB/GBL, are highly addictive and the use of these drugs for chemsex may lead to more extensive drug use, including injectable substances and drug dependency (Drückler et al., 2018, Uholyeva and Pitoňák, 2022) often with serious socio-economic and health implications at both an individual and societal level (GBD, 2016 Alcohol and Drug Use Collaborators, 2018).

The fact that as many as 50 % of the respondents reported some drug use as measured by the DUDIT instrument was partly due to the frequent use of poppers among our participants (56 %) classified as a drug in this instrument (Berman et al., 2005). Poppers are not illegal in Sweden, and common at both parties and sexual encounters among MSM, and often perceived by the group as relatively unproblematic. We did not find any significant correlation between the use of poppers and sexual risk behaviors among MSM. Given how prevalent poppers use is among MSM, it dilutes the specificity of the definition and assessment of chemsex. If poppers use alone qualifies as chemsex, many individuals are categorized as engaging in chemsex despite not exhibiting other substance use or risk behaviors. Moreover, the data shows no correlation between poppers use and sexual risk behavior, such as condomless sex. This is in contrast with the narrower definition of chemsex (e.g., including GHB, methamphetamine, and/or mephedrone), which is clearly associated with such behaviors. Also, 13 % of the respondents were classified as having probable drug addiction. This underscores the importance for sexual health services, PrEP providers, and social workers, to identify and address the need for support associated with problematic drug use among MSM.

Consistent with our findings, others have shown that chemsex is associated with high alcohol consumption (Maxwell et al., 2019, Bohn et al., 2020, Scholz-Hehn et al., 2022). The Swedish MSM cohort on PrEP reported a much higher alcohol consumption than the general population, with 26 % of the study participants reporting risky use of alcohol according to AUDIT, as compared to 16 % of Swedes in general (Public Health Agency of Sweden, 2023).

Our study addresses an important gap in understanding chemsex among MSM in Sweden. Since the rollout of PrEP for HIV prevention in 2018, significant public health benefits have been observed (Hansson et al., 2020). By reducing the fear of HIV transmission, PrEP has also contributed to increased sexual risk-taking (Price et al., 2022). Previous studies show that MSM who engage in chemsex are more likely to report multiple and concurrent sexual partners, often during the same sexual encounter (Guerra et al., 2020, Wang et al., 2023). Our findings confirm that respondents using chemsex had significantly more casual partners, more frequent group sex, and higher rates of condomless anal sex. This suggests a strong link between drug use and elevated sexual risk-taking, likely intensified by the disinhibiting effects of substances used in chemsex (Poulios et al., 2022). These patterns are consistent with data from other European countries (Maxwell et al., 2019, De La Mora et al., 2023). More partners in turn raises the likelihood of acquiring other STIs (Poulios et al., 2022, Amundsen et al., 2022), but more frequent HIV testing and greater sexual health awareness among those on PrEP also likely contribute to the increased detection of STIs (Stewart and Baeten, 2022, Hovaguimian et al., 2024). Thus, although consistent PrEP use offers strong protection against HIV, chemsex-associated behaviors—especially involving multiple partners—can increase STI transmission risk and pose a threat to individuals who are not on PrEP participating in the same sexual networks (Stewart, Baeten, 2022). Regardless, this highlights the public health relevance of discussing behaviors like multiple concurrent partnerships, group sex, and condomless intercourse—even in populations with low HIV risk.

Beyond enhancing sexual experiences, drug use in sexual contexts is also employed as a coping mechanism to manage minority stress, HIV-related stigma, and internalized homophobia (Poulios et al., 2022). The stigma and discrimination often faced by MSM can lead to shame and low self-worth, which some attempt to alleviate through drug use during sex. This strategy becomes problematic when individuals lose control over their use or rely solely on drugs to cope with identity or sexual issues, potentially leading to dependence and heightened sexual risk behaviors (Poulios et al., 2022, Dubov et al., 2018). Moreover, the normalization of drug use in certain MSM social settings may obscure the risks of chemsex, particularly for younger or socio-economically vulnerable MSM, and reduce the ability to recognize the risks associated with chemsex (Maxwell et al., 2019, De La Mora et al., 2023). In party settings—whether at home or in clubs—chemsex drugs are often readily available, and social pressure can influence individuals to participate even if they had not intended to do so (Evers et al., 2020).

Many MSM engaging in chemsex express a preference for counseling by sexual health professionals, particularly regarding issues such as maintaining self-control (Evers et al., 2020b). Enhancing professional knowledge of chemsex, including the associated risks and effective interventions, may improve the ability of healthcare providers and social workers to identify problematic drug use and offer appropriate support.

Since chemsex is not necessarily problematic for all individuals, health care professionals and social workers should investigate whether individuals themselves perceive their chemsex use as an issue, while being careful to avoid further stigmatization of already marginalized groups. When drug use is identified as problematic, harm reduction strategies—such as providing information on substance combinations and emphasizing the importance of hydration and sleep—may be appropriate (Chemsexharmreduction.org.org, 2025). In cases where chemsex use has resulted in substance addiction, referral to specialized addiction treatment may be necessary. Unlike several other European countries that have decriminalized or legalized certain drugs, Sweden maintains strict laws against all illegal drug use. Possession and consumption of even small amounts of drugs, including chemsex-related drugs, can result in legal consequences, including fines and imprisonment (SFS, 1968:649). Something that not all people who use drugs may be aware of, but these legal implications could limit their willingness to seek care and harm reduction.

### 4.1 Limitations

Certain limitations of this study should be mentioned. First, the population for the study is limited to MSM on PrEP and therefore excludes other MSM such as those living with HIV and other MSM not on PrEP. Therefore, findings may not generalize to the wider MSM population. Also, the questionnaire was only available online and in Swedish and English, excluding any patients without access to smartphones or computers and those in need of a translator. This may imply that the results do not reflect the conditions for, for example, asylum seekers, socioeconomically disadvantaged individuals, and other vulnerable groups.

## 5 Conclusions

In conclusion, one in five respondents among MSM on PrEP in Stockholm engage in chemsex, in turn associated with high alcohol consumption, addictive drug use and increased sexual risk behaviors such as condomless anal sex, group sex and multiple casual partners.

Differentiating between types of substance use and their links to sexual risk can guide more effective and targeted interventions, where a broader definition of chemsex likely captures a lower-risk population. These findings highlight the imperative to develop systematic approaches for identifying MSM who participate in chemsex and are in need of evidence-based, individualized interventions and clinical support. By focusing on harm reduction, mental health, social support, and advocacy, health professionals and social workers can significantly improve the lives of MSM with problematic substance use and contribute to reducing health disparities in this population.

While our findings provide novel insights into the situation for MSM in Sweden, the study also highlights the need for more research on the use of chemsex and its implications in other groups within the MSM community, transgender individuals, and the impact on younger age groups and MSM living with HIV.

## CRediT authorship contribution statement

**Westergren Victor:** Writing – review & editing, Visualization, Conceptualization. **Rost Elin:** Writing – review & editing, Writing – original draft, Visualization, Investigation, Formal analysis, Conceptualization. **Ekström Anna Mia:** Writing – review & editing, Supervision, Resources, Project administration, Conceptualization. **Luksha Yauheni:** Writing – review & editing. **Lindberg Daniel Per:** Writing – review & editing, Supervision, Formal analysis.

## Funding

The Public Health Agency of Sweden.

## Declaration of Competing Interest

The authors declare that they have no known competing financial interests or personal relationships that could have appeared to influence the work reported in this paper.
